# Methyl 2-(4-chloro­benzamido)­benzoate

**DOI:** 10.1107/S160053681004897X

**Published:** 2010-11-27

**Authors:** Islam Ullah Khan, Onur Şahin, Rashid Javaid, Shahzad Sharif, Orhan Büyükgüngör

**Affiliations:** a Materials Chemistry Laboratory, Department of Chemistry, Government College University, Lahore 54000, Pakistan; b Department of Physics, Ondokuz Mayıs University, TR-55139 Samsun, Turkey

## Abstract

In the title compound, C_15_H_12_ClNO_3_, the central C—C(O)—N—C amide unit makes dihedral angles of 6.60 (2) and 3.42 (2)°, respectively, with the 4-chloro­benzene and anilino rings. The dihedral angle between the two benzene rings is 3.32 (3)°. Intra­molecular N—H⋯O and C—H⋯O hydrogen bonds form *S*(6) rings and contribute to the planarity of this portion of the mol­ecule. In the crystal, inter­molecular C—H⋯O hydrogen bonds are observed, which link the mol­ecules into [010] *C*(7) chains.

## Related literature

For the graph-set analysis of hydrogen-bond patterns, see: Bernstein *et al.* (1995 ▶). For related structures, see: Gowda *et al.* (2008 ▶); Zhou & Zheng (2007 ▶); Khan *et al.* (2010 ▶). Benzamide derivatives are frequently used in the synthesis of new and effective anti-convulsant agents, see: Clark *et al.* (1988 ▶); Leander *et al.* (1988 ▶); Diouf *et al.* (1997 ▶). For bond-length data, see: Allen *et al.* (1987 ▶).
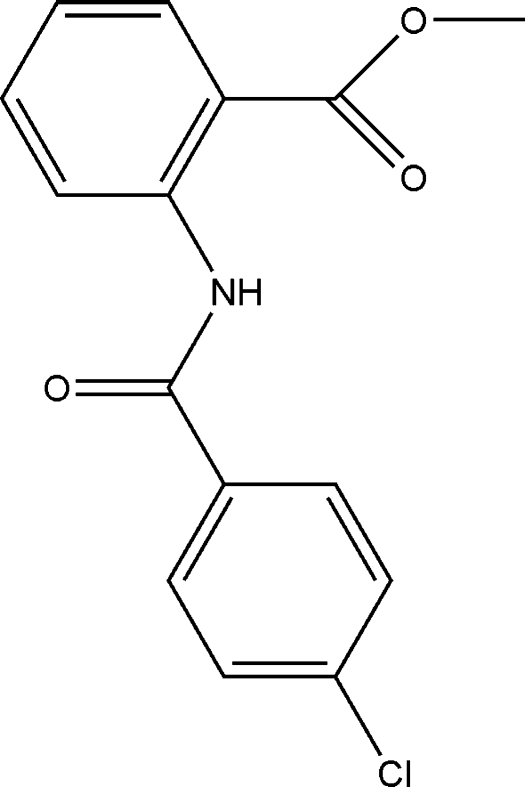


## Experimental

### 

#### Crystal data

C_15_H_12_ClNO_3_
*M* *_r_* = 289.71
Orthorhombic, 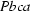

*a* = 7.3788 (9) Å
*b* = 16.757 (2) Å
*c* = 21.530 (2) Å
*V* = 2662.0 (5) Å^3^
*Z* = 8
Mo *K*α radiation
μ = 0.29 mm^−1^
*T* = 296 K
0.21 × 0.12 × 0.08 mm

#### Data collection

Bruker SMART APEXII diffractometer
11139 measured reflections
2399 independent reflections
814 reflections with *I* > 2σ(*I*)
*R*_int_ = 0.166

#### Refinement

*R*[*F* ^2^ > 2σ(*F* ^2^)] = 0.059
*wR*(*F* ^2^) = 0.134
*S* = 0.97
2399 reflections
182 parameters
H-atom parameters constrained
Δρ_max_ = 0.21 e Å^−3^
Δρ_min_ = −0.26 e Å^−3^

### 

Data collection: *APEX2* (Bruker, 2007 ▶); cell refinement: *SAINT* (Bruker, 2007 ▶); data reduction: *SAINT*; program(s) used to solve structure: *SHELXS97* (Sheldrick, 2008 ▶); program(s) used to refine structure: *SHELXL97* (Sheldrick, 2008 ▶); molecular graphics: *ORTEP-3 for Windows* (Farrugia, 1997 ▶); software used to prepare material for publication: *WinGX* (Farrugia, 1999 ▶).

## Supplementary Material

Crystal structure: contains datablocks global, I. DOI: 10.1107/S160053681004897X/om2376sup1.cif

Structure factors: contains datablocks I. DOI: 10.1107/S160053681004897X/om2376Isup2.hkl

Additional supplementary materials:  crystallographic information; 3D view; checkCIF report

## Figures and Tables

**Table 1:** Hydrogen-bond geometry (Å, °)

*D*—H⋯*A*	*D*—H	H⋯*A*	*D*⋯*A*	*D*—H⋯*A*
C2—H2⋯O1	0.93	2.21	2.839 (6)	124
N1—H1⋯O2	0.86	1.94	2.646 (5)	138
C3—H3⋯O2^i^	0.93	2.58	3.422 (6)	151

Symmetry code: (i) 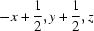.

**Fig. 1.**
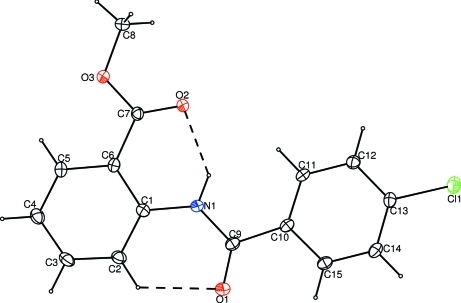
A view of the title compound showing displacement ellipsoids drawn at the 30% probability level. Hydrogen bonds are indicated by dashed lines.

**Fig. 2.**
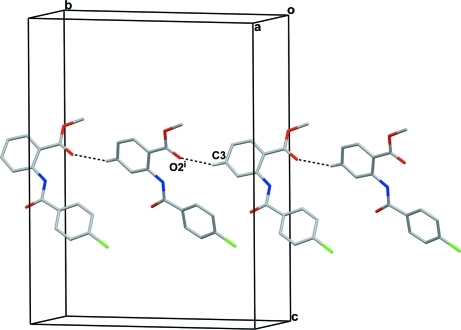
Part of the crystal structure, showing the formation of a C(7) chain running parallel to the [010] direction. For the sake of clarity, H atoms not involved in the motif shown have been omitted (symmetry code: i = -x+1/2, y+1/2, z)).

## supplementary crystallographic information

### Comment

Benzamide derivatives are frequently used in the synthesis of new and effective anti-convulsant agents (Clark *et al.*, 1988; Leander *et al.*, 1988; Diouf *et al.*, 1997). In continuation of our ongoing structural studies of benzamide derivatives (Khan *et al.*, 2010), herein the crystal structure of title compound is described.

In the title compound, Fig. 1, the C1—N1—C9(O1)—C10 amide unit is planar, r.m.s. deviation 0.0102 Å, and subtends dihedral angles of 3.42 (2)° and 6.60 (2)° respectively to the C1···C6 and C10···C15 rings. The two aromatic rings are inclined at 3.32 (3)°. Bond distances within the molecule are normal (Allen *et al.* 1987) and similar to those observed in comparable structures (Gowda *et al.* 2008; Zhou & Zheng 2007).

The intramolecular C2—H2···O1 and N1—H1···O2 hydrogen bonds produce S(6) rings (Bernstein *et al.*, 1995) (Fig. 1). The C3—H3 group in the molecule at acts as a hydrogen-bond donor to atom O2^i^ (symmetry code: -x+1/2, y+1/2, z) forming a C(7) chain running parallel to the [010] direction (Fig. 2).

### Experimental

A solution of methyl anthranilate (390 µl, 3 mmol) in dichloromethane (15 ml) was treated dropwise with 4-chlorobenzoyl chloride (383 µl, 3 mmol) in the presence of triethanolamine (5 ml) as a catalyst. The resulting mixture was stirred for 1 h. On completion of reaction, precipitates formed, were filtered, dried and crystallized from methanol to yield colorless blocks of the title compound.

### Refinement

All H-atoms were refined using riding model for hydrogen bonds with d(C—H) = 0.93Å (*U*_iso_=1.2*U*_eq_ of the parent atom) for aromatic carbon atoms, d(N—H) = 0.86Å (*U*_iso_=1.2*U*_eq_ of the parent atom) for imine nitrogen atom and d(C—H) = 0.96Å (*U*_iso_=1.5*U*_eq_ of the parent atom) for methyl carbon atom.

### Crystal data

**Table:**

C_15_H_12_ClNO_3_	*F*(000) = 1200
*M**_r_* = 289.71	*D*_x_ = 1.446 Mg m^−^^3^
Orthorhombic, *P**b**c**a*	Mo *K*α radiation, λ = 0.71073 Å
Hall symbol: -P 2ac 2ab	Cell parameters from 11747 reflections
*a* = 7.3788 (9) Å	θ = 3.1–16.8°
*b* = 16.757 (2) Å	µ = 0.29 mm^−^^1^
*c* = 21.530 (2) Å	*T* = 296 K
*V* = 2662.0 (5) Å^3^	Block, colorless
*Z* = 8	0.21 × 0.12 × 0.08 mm

### Data collection

**Table:**

Bruker SMART APEXII diffractometer	814 reflections with *I* > 2σ(*I*)
Radiation source: fine-focus sealed tube	*R*_int_ = 0.166
graphite	θ_max_ = 25.2°, θ_min_ = 3.1°
φ and ω scans	*h* = −9→5
11139 measured reflections	*k* = −20→20
2399 independent reflections	*l* = −26→23

### Refinement

**Table:**

Refinement on *F*^2^	Primary atom site location: structure-invariant direct methods
Least-squares matrix: full	Secondary atom site location: difference Fourier map
*R*[*F*^2^ > 2σ(*F*^2^)] = 0.059	Hydrogen site location: inferred from neighbouring sites
*wR*(*F*^2^) = 0.134	H-atom parameters constrained
*S* = 0.97	*w* = 1/[σ^2^(*F*_o_^2^) + (0.0301*P*)^2^] where *P* = (*F*_o_^2^ + 2*F*_c_^2^)/3
2399 reflections	(Δ/σ)_max_ < 0.001
182 parameters	Δρ_max_ = 0.21 e Å^−^^3^
0 restraints	Δρ_min_ = −0.26 e Å^−^^3^

### Special details

**Table:**

Geometry. All esds (except the esd in the dihedral angle between two l.s. planes) are estimated using the full covariance matrix. The cell esds are taken into account individually in the estimation of esds in distances, angles and torsion angles; correlations between esds in cell parameters are only used when they are defined by crystal symmetry. An approximate (isotropic) treatment of cell esds is used for estimating esds involving l.s. planes.
Refinement. Refinement of *F*^2^ against ALL reflections. The weighted *R*-factor *wR* and goodness of fit *S* are based on *F*^2^, conventional *R*-factors *R* are based on *F*, with *F* set to zero for negative *F*^2^. The threshold expression of *F*^2^ > σ(*F*^2^) is used only for calculating *R*-factors(gt) *etc*. and is not relevant to the choice of reflections for refinement. *R*-factors based on *F*^2^ are statistically about twice as large as those based on *F*, and *R*- factors based on ALL data will be even larger.

### Fractional atomic coordinates and isotropic or equivalent isotropic displacement parameters (Å^2^)

**Table:**

	*x*	*y*	*z*	*U*_iso_*/*U*_eq_	
C1	0.2912 (7)	0.0979 (3)	0.5068 (2)	0.0455 (14)	
C2	0.3378 (8)	0.1763 (3)	0.5195 (2)	0.0592 (16)	
H2	0.3927	0.1892	0.5571	0.071*	
C3	0.3031 (8)	0.2348 (3)	0.4770 (3)	0.0663 (17)	
H3	0.3357	0.2872	0.4857	0.080*	
C4	0.2209 (7)	0.2174 (3)	0.4215 (3)	0.0637 (17)	
H4	0.1955	0.2580	0.3933	0.076*	
C5	0.1760 (7)	0.1399 (3)	0.4076 (2)	0.0533 (16)	
H5	0.1213	0.1281	0.3698	0.064*	
C6	0.2116 (7)	0.0788 (3)	0.4498 (2)	0.0425 (14)	
C7	0.1648 (7)	−0.0040 (3)	0.4327 (2)	0.0487 (16)	
C8	0.0594 (7)	−0.0888 (3)	0.3536 (2)	0.0757 (19)	
H8A	0.1720	−0.1136	0.3424	0.113*	
H8B	−0.0180	−0.0861	0.3178	0.113*	
H8C	0.0013	−0.1196	0.3855	0.113*	
C9	0.3924 (7)	0.0410 (3)	0.6083 (2)	0.0522 (16)	
C10	0.4046 (7)	−0.0374 (3)	0.6420 (2)	0.0447 (14)	
C11	0.3391 (7)	−0.1095 (3)	0.6209 (2)	0.0515 (16)	
H11	0.2803	−0.1118	0.5827	0.062*	
C12	0.3590 (7)	−0.1781 (3)	0.6552 (2)	0.0559 (16)	
H12	0.3158	−0.2265	0.6401	0.067*	
C13	0.4442 (7)	−0.1741 (3)	0.7124 (2)	0.0523 (15)	
C14	0.5107 (7)	−0.1034 (3)	0.7343 (2)	0.0571 (16)	
H14	0.5693	−0.1013	0.7725	0.069*	
C15	0.4904 (7)	−0.0356 (3)	0.6994 (2)	0.0557 (16)	
H15	0.5349	0.0126	0.7145	0.067*	
N1	0.3251 (5)	0.0360 (2)	0.54958 (18)	0.0502 (12)	
H1	0.2999	−0.0113	0.5368	0.060*	
O1	0.4375 (6)	0.1027 (2)	0.63322 (15)	0.0849 (14)	
O2	0.1871 (5)	−0.06159 (18)	0.46533 (15)	0.0636 (12)	
O3	0.0940 (5)	−0.00921 (19)	0.37634 (15)	0.0615 (11)	
Cl1	0.4671 (2)	−0.25964 (8)	0.75727 (6)	0.0747 (6)	

### Atomic displacement parameters (Å^2^)

**Table:**

	*U*^11^	*U*^22^	*U*^33^	*U*^12^	*U*^13^	*U*^23^
C1	0.043 (4)	0.042 (3)	0.051 (4)	0.003 (3)	0.009 (3)	−0.003 (3)
C2	0.073 (5)	0.050 (4)	0.054 (4)	−0.003 (3)	0.004 (3)	−0.004 (3)
C3	0.085 (5)	0.041 (3)	0.073 (4)	−0.003 (4)	0.008 (4)	−0.006 (3)
C4	0.070 (5)	0.051 (4)	0.070 (4)	0.013 (4)	0.012 (4)	0.008 (3)
C5	0.054 (5)	0.052 (4)	0.054 (4)	0.004 (3)	0.004 (3)	0.006 (3)
C6	0.039 (4)	0.047 (3)	0.041 (3)	0.001 (3)	0.005 (3)	0.005 (3)
C7	0.048 (5)	0.054 (4)	0.044 (4)	0.000 (4)	0.001 (3)	0.004 (3)
C8	0.110 (6)	0.056 (4)	0.061 (4)	−0.006 (4)	−0.022 (4)	−0.008 (3)
C9	0.054 (5)	0.058 (4)	0.045 (4)	0.001 (3)	−0.001 (3)	−0.008 (3)
C10	0.042 (4)	0.060 (4)	0.033 (3)	0.003 (3)	0.003 (3)	−0.006 (3)
C11	0.057 (5)	0.061 (4)	0.037 (3)	−0.001 (3)	−0.010 (3)	−0.001 (3)
C12	0.064 (5)	0.055 (4)	0.049 (4)	0.008 (3)	0.000 (3)	−0.008 (3)
C13	0.054 (5)	0.056 (4)	0.047 (4)	0.014 (3)	0.010 (3)	0.006 (3)
C14	0.054 (5)	0.075 (4)	0.042 (3)	0.006 (4)	−0.003 (3)	−0.002 (3)
C15	0.059 (5)	0.065 (4)	0.043 (4)	−0.005 (3)	0.006 (3)	−0.009 (3)
N1	0.060 (4)	0.046 (3)	0.044 (3)	−0.002 (2)	−0.006 (2)	−0.002 (2)
O1	0.132 (4)	0.061 (3)	0.062 (3)	−0.015 (3)	−0.023 (2)	−0.008 (2)
O2	0.094 (4)	0.043 (2)	0.054 (2)	−0.005 (2)	−0.020 (2)	0.0071 (18)
O3	0.085 (4)	0.049 (2)	0.051 (2)	−0.006 (2)	−0.015 (2)	0.0026 (18)
Cl1	0.0844 (13)	0.0744 (10)	0.0654 (9)	0.0159 (10)	−0.0047 (9)	0.0112 (8)

### Geometric parameters (Å, °)

**Table:**

C1—C2	1.386 (6)	C8—H8C	0.9600
C1—C6	1.398 (6)	C9—O1	1.212 (5)
C1—N1	1.410 (5)	C9—N1	1.360 (5)
C2—C3	1.365 (6)	C9—C10	1.503 (6)
C2—H2	0.9300	C10—C11	1.379 (6)
C3—C4	1.371 (6)	C10—C15	1.388 (6)
C3—H3	0.9300	C11—C12	1.373 (6)
C4—C5	1.373 (6)	C11—H11	0.9300
C4—H4	0.9300	C12—C13	1.385 (6)
C5—C6	1.393 (6)	C12—H12	0.9300
C5—H5	0.9300	C13—C14	1.367 (6)
C6—C7	1.476 (6)	C13—Cl1	1.737 (5)
C7—O2	1.206 (5)	C14—C15	1.370 (6)
C7—O3	1.323 (5)	C14—H14	0.9300
C8—O3	1.443 (5)	C15—H15	0.9300
C8—H8A	0.9600	N1—H1	0.8600
C8—H8B	0.9600		
			
C2—C1—C6	119.7 (5)	H8B—C8—H8C	109.5
C2—C1—N1	121.6 (5)	O1—C9—N1	124.3 (5)
C6—C1—N1	118.7 (4)	O1—C9—C10	121.0 (5)
C3—C2—C1	120.1 (5)	N1—C9—C10	114.7 (5)
C3—C2—H2	120.0	C11—C10—C15	118.2 (5)
C1—C2—H2	120.0	C11—C10—C9	125.8 (5)
C2—C3—C4	121.0 (5)	C15—C10—C9	116.0 (5)
C2—C3—H3	119.5	C12—C11—C10	121.3 (5)
C4—C3—H3	119.5	C12—C11—H11	119.4
C3—C4—C5	119.8 (5)	C10—C11—H11	119.4
C3—C4—H4	120.1	C11—C12—C13	119.1 (5)
C5—C4—H4	120.1	C11—C12—H12	120.5
C4—C5—C6	120.5 (5)	C13—C12—H12	120.5
C4—C5—H5	119.7	C14—C13—C12	120.8 (5)
C6—C5—H5	119.7	C14—C13—Cl1	119.3 (4)
C5—C6—C1	118.9 (5)	C12—C13—Cl1	120.0 (5)
C5—C6—C7	119.0 (5)	C13—C14—C15	119.4 (5)
C1—C6—C7	122.2 (5)	C13—C14—H14	120.3
O2—C7—O3	122.3 (5)	C15—C14—H14	120.3
O2—C7—C6	125.1 (5)	C14—C15—C10	121.3 (5)
O3—C7—C6	112.5 (4)	C14—C15—H15	119.4
O3—C8—H8A	109.5	C10—C15—H15	119.4
O3—C8—H8B	109.5	C9—N1—C1	128.8 (4)
H8A—C8—H8B	109.5	C9—N1—H1	115.6
O3—C8—H8C	109.5	C1—N1—H1	115.6
H8A—C8—H8C	109.5	C7—O3—C8	116.2 (4)
			
C6—C1—C2—C3	1.1 (8)	N1—C9—C10—C15	173.4 (4)
N1—C1—C2—C3	179.9 (5)	C15—C10—C11—C12	−0.6 (8)
C1—C2—C3—C4	0.6 (9)	C9—C10—C11—C12	179.3 (5)
C2—C3—C4—C5	−1.5 (9)	C10—C11—C12—C13	1.0 (8)
C3—C4—C5—C6	0.7 (9)	C11—C12—C13—C14	−1.1 (8)
C4—C5—C6—C1	1.0 (8)	C11—C12—C13—Cl1	178.7 (4)
C4—C5—C6—C7	−178.8 (5)	C12—C13—C14—C15	0.9 (8)
C2—C1—C6—C5	−1.9 (8)	Cl1—C13—C14—C15	−179.0 (4)
N1—C1—C6—C5	179.3 (4)	C13—C14—C15—C10	−0.5 (8)
C2—C1—C6—C7	177.8 (5)	C11—C10—C15—C14	0.3 (8)
N1—C1—C6—C7	−1.0 (7)	C9—C10—C15—C14	−179.6 (5)
C5—C6—C7—O2	−179.1 (5)	O1—C9—N1—C1	−1.1 (9)
C1—C6—C7—O2	1.1 (8)	C10—C9—N1—C1	177.8 (4)
C5—C6—C7—O3	0.9 (7)	C2—C1—N1—C9	4.7 (8)
C1—C6—C7—O3	−178.8 (5)	C6—C1—N1—C9	−176.5 (5)
O1—C9—C10—C11	172.5 (5)	O2—C7—O3—C8	−5.4 (7)
N1—C9—C10—C11	−6.5 (7)	C6—C7—O3—C8	174.6 (4)
O1—C9—C10—C15	−7.7 (8)		

### Hydrogen-bond geometry (Å, °)

**Table:**

*D*—H···*A*	*D*—H	H···*A*	*D*···*A*	*D*—H···*A*
C2—H2···O1	0.93	2.21	2.839 (6)	124
N1—H1···O2	0.86	1.94	2.646 (5)	138
C3—H3···O2^i^	0.93	2.58	3.422 (6)	151

Symmetry codes: (i) −*x*+1/2, *y*+1/2, *z*.
